# A Review of Graphene-Based Materials/Polymer Composite Aerogels

**DOI:** 10.3390/polym15081888

**Published:** 2023-04-14

**Authors:** Ze Wang, Libao Liu, Yiwei Zhang, Yi Huang, Jia Liu, Xu Zhang, Xu Liu, Huaibao Teng, Xiaofang Zhang, Jianming Zhang, Hongsheng Yang

**Affiliations:** Key Laboratory of Rubber-Plastics, Ministry of Education/Shandong Provincial Key Laboratory of Rubber-Plastics, School of Polymer Science and Engineering, Qingdao University of Science & Technology, Qingdao 266042, China; sunshine20230327@163.com (Z.W.); liulibaovb@163.com (L.L.); zhangyiwei19981001@163.com (Y.Z.); hy1721102606@163.com (Y.H.); m19805446216@163.com (J.L.); zx13853015859@163.com (X.Z.); s15020482080@163.com (X.L.); 15335421706@163.com (H.T.); xfzhangyuer@126.com (X.Z.); zjm@qust.edu.cn (J.Z.)

**Keywords:** graphene aerogels, polymer composites, preparation methods, properties, applications

## Abstract

The fabrication of composite materials is an effective way to improve the performance of a single material and expand its application range. In recent years, graphene-based materials/polymer composite aerogels have become a hot research field for preparing high-performance composites due to their special synergistic effects in mechanical and functional properties. In this paper, the preparation methods, structures, interactions, properties, and applications of graphene-based materials/polymer composite aerogels are discussed, and their development trend is projected. This paper aims to arouse extensive research interests in multidisciplinary fields and provide guidance for the rational design of advanced aerogel materials, which could then encourage efforts to use these new kinds of advanced materials in basic research and commercial applications.

## 1. Introduction

Aerogels are a kind of ultralight nanoporous material with three-dimensional (3D) network structures. Benefitting from its ultralow density, ultrahigh porosity, specific surface area, excellent mass transfer property, and ultralow thermal conductivity, the aerogel shows great promise in numerous fields, such as high adsorption [1], thermal insulation [2], sensors [3], and energy storage [4]. Polymer foams have excellent comprehensive properties, such as light weight and high porosity, and are widely used in packaging, heat insulation, and sound absorption in daily life. Polymer foams are one of many different types of aerogels. However, due to large pore size and lack of functionality, its application range is limited. Graphene-based aerogels are a kind of 3D metamaterial with a highly porous structure assembled by two-dimensional graphene-based nanomaterials. Among them, graphene-based materials include many derivative materials, such as graphene nanosheets (GNS), graphene oxide (GO), reduced graphene oxide (RGO), and so on [5]. In this paper, the graphene aerogels refer to all aerogels prepared by the graphene-related derivative materials unless otherwise specified. Graphene aerogels have attracted much attention because of their high porosity (>99%) [6,7,8,9], low density (<10 mg/cm^3^), high elasticity (compressive resilience strain up to 99%) in a wide temperature range (−196 °C to 1000 °C) [10,11,12,13,14,15,16,17,18], and excellent photothermal and electrothermal conversion ability [19,20,21,22]. However, graphene aerogels also have some disadvantages. For example, they have low mechanical strength and poor flexibility. Compositing graphene-based materials with polymer to prepare composite aerogels is an effective strategy to solve the above problems. For example, the introduction of polymer can improve the poor mechanical properties of pure graphene aerogels [23,24]. Similarly, the polymer foams can be endowed with multiple functions, such as good electromagnetic shielding performance and the possibility of application in compressible batteries after blending with graphene-based materials [25,26].

**Figure 1:**
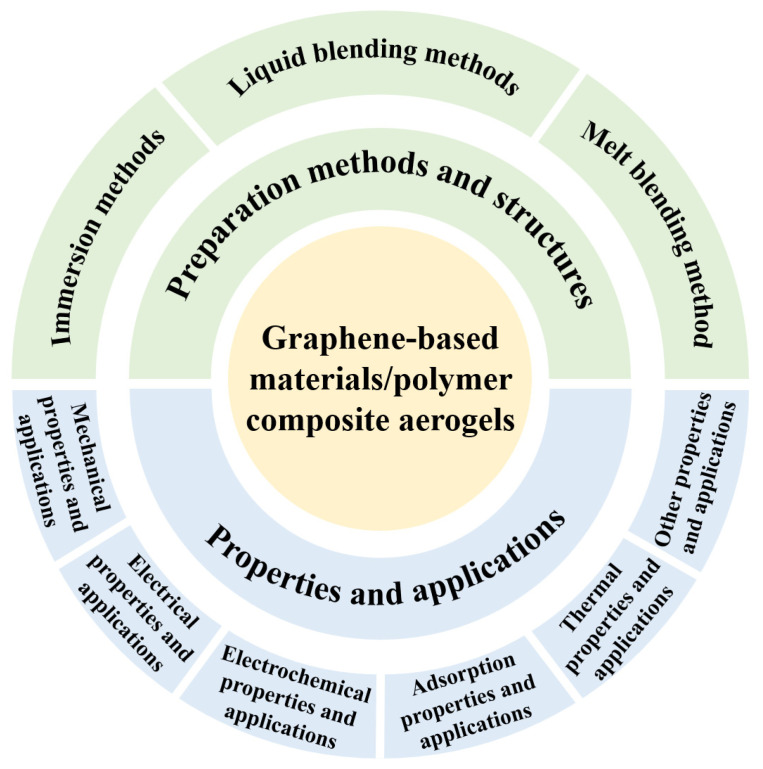
The guide map of this review on graphene-based materials/polymer composite aerogels.

In recent years, graphene-based materials/polymer composite aerogels have been extensively studied. As shown in Figure 1, this paper summarizes the preparation methods, structures, interactions, properties, applications of graphene-based materials/polymer composite aerogels. First, the preparation methods are discussed and divided into three categories, including impregnation methods, liquid blending methods, and melt blending methods. Second, the structures and interactions of graphene-based materials/polymer composite aerogels are discussed. Finally, the mechanical properties, electrical properties, thermal properties, adsorption properties, other properties, and the respective applications of graphene-based materials/polymer composite aerogels were discussed. This paper will both help readers quickly gain familiarity with this field and provide them with more guidance about the rational design and preparation of advanced aerogel materials.

## 2. Preparations and Structures of Graphene-Based Materials/Polymer Composite Aerogels

**Figure 2:**
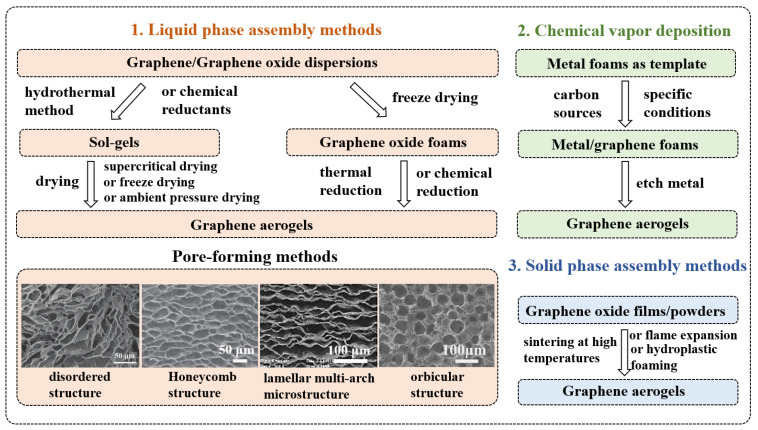
Preparation methods summary of graphene aerogels.

**Figure 3:**
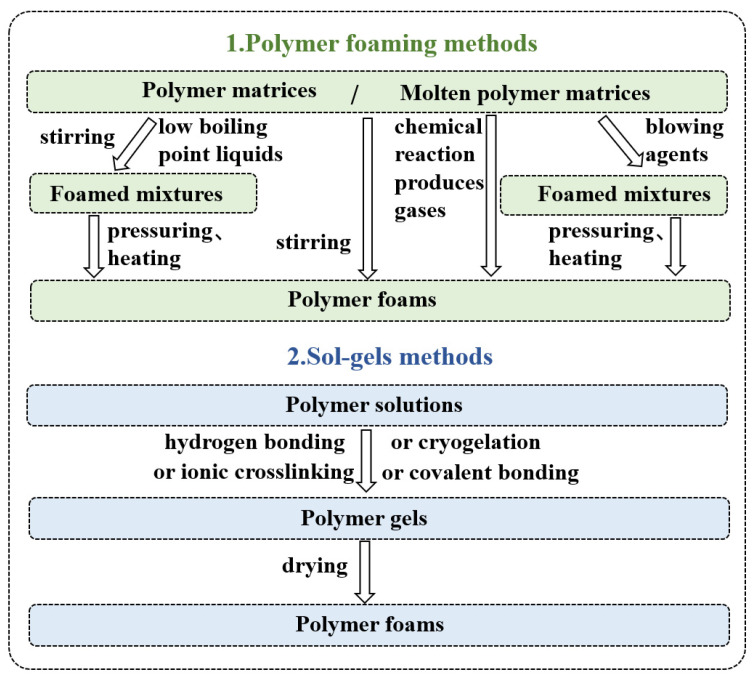
Preparation methods summary of polymer foams.

In order to better understand the composition of graphene-based materials/polymer composite aerogels, Figure 2 and Figure 3 summarize the preparation methods of graphene aerogels and polymer foams, respectively. The preparation methods of graphene aerogels are mainly divided into three categories, including liquid phase assembly [7], chemical vapor deposition (CVD) [6], and solid phase assembly [27]. The liquid phase assembly method is mainly divided into two categories according to the preparation process. The first method is as follows. GO solution formed a wet gel (sol-gel) using the hydrothermal [7] or chemical method [28,29,30]. Then, graphene aerogels were obtained by drying through various techniques. In the second method, GO solutions were directly freeze-dried [13], then graphene aerogels were obtained using chemical vapor reduction or thermal reduction [31,32]. The liquid phase assembly method involves pore formation and drying. Ice templates [12,33,34,35,36,37], bubble templates [11,38,39,40], or emulsion templates [22,41,42] are commonly used to regulate the pore structures. The SEM images of graphene aerogels with different pore structures are shown in Figure 2. At present, the drying methods include supercritical drying [8,43,44,45,46], freeze drying [47,48,49,50,51], and ambient pressure drying [10,14,15,52,53]. The CVD template method involves depositing graphene on the metal foam substrates through chemical vapor deposition, then etching the metal foam substrates [54,55,56]. Finally, solid phase assembly methods mainly use GO films (for flame expansion or plastic foaming) or powders (for high temperature welding) as precursors to prepare graphene aerogels. [57,58].

Polymer foams are mainly prepared using polymer foaming or gel-drying methods. Polymer foaming methods are divided into three types according to the foaming method: physical, mechanical, and chemical. In physical foaming, low-boiling-point (BP) liquid and polymer are blended and then foamed through pressuring and heating [59]. In addition, air is added in polymer resin by mechanical stirring; then, the resin is foamed. Finally, the chemical foaming process can be undertaken using the following methods. In the first method, chemical reactions between the two polymers occur to produce inert gases, and then, the polymers are foamed. In the second method, a foaming agent is added to molten polymer, and a gas is released. Then, the polymer is foamed by pressure and heating [60]. Physical foaming is the most widely used method. Because the foaming agent used in the physical foaming method is generally inert compressed gas, it only changes in the physical form during the foaming process, making the physical foaming method greener and more environmentally friendly. There is no chemical residue in the prepared polymer foam material, which can meet people’s increasing requirements for polymer foam materials.

### 2.1. Immersion Methods

The impregnation method includes two steps. First, the 3D network skeleton is prepared in advance, and then, the composite is immersed into the 3D skeleton under vacuum or hydrophobic action. The composite material is obtained after curing.

#### 2.1.1. Graphene-Based Materials Aerogels Impregnated in Polymer Solutions

**Figure 4:**
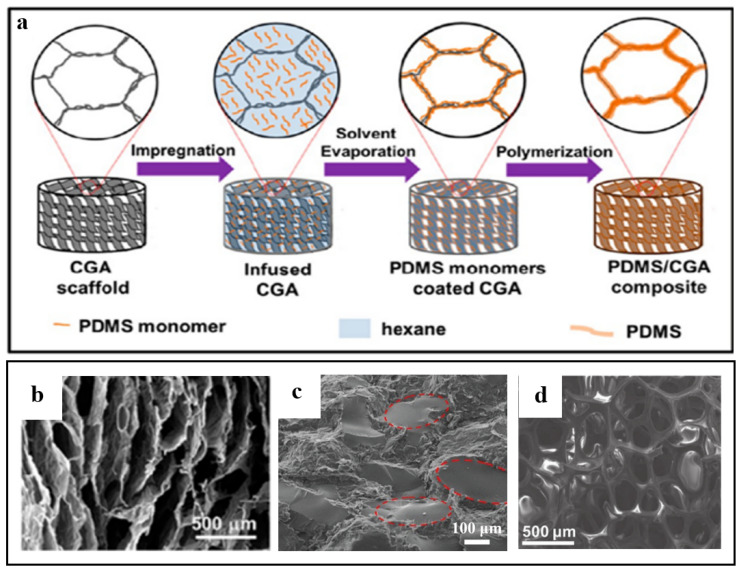
(**a**) Schematic of the procedure for fabricating graphene/PDMS aerogel. (**b**) The SEM image of graphene/PDMS aerogel. Reproduced with permission from [61]. (**c**) Microstructure of 3D GNPS/RGO foam/epoxy nanocomposites. Reproduced with permission from [62]. (**d**) The SEM image of graphene/PDMS foam composites. Reproduced with permission from [63].

As shown in Figure 4a, Hu et al. [61] first prepared compressible graphene aerogel (CGA) with porous structure. Subsequently, it was immersed in a hexane solution containing poly(dimethylsiloxane) (PDMS) monomer and curing agent, and then placed in a vacuum furnace and heated to remove the solvent. Further heating-induced PDMS was polymerized on the cell walls of graphene aerogel, and finally, graphene/PDMS composite aerogel was obtained. The graphene/PDMS composite aerogel exhibited a 3D interconnected structure, indicating that PDMS was mainly distributed on the surface of the cell wall instead of in the filling hole (Figure 4b). Liang et al. [62] combined 3D graphene nanoplatelets (GNPS)/RGO foam with epoxy using the impregnation method and prepared the composite material. Figure 4c shows that the cured epoxy resin was evenly distributed in the micropore space. The size of the spherical block is between 50~150 μm, which is consistent with the pore size of the GNPS/RGO foam skeleton; this demonstrates that the structure of the GNPS/RGO foam skeleton is inherited after filling the epoxy resin. In addition, Chen et al. [63] grew graphene on nickel foam by CVD at 1000 °C and atmospheric pressure, which was similar to the structure of nickel foam. Then, a thin layer of PDMS was impregnated on the surface of the graphene, and the graphene/PDMS composite foam was obtained by etching the nickel foam substrate with HCl. As shown in Figure 4d, the composite has high porosity and inherits the 3D network structure of nickel foam well.

#### 2.1.2. Graphene-Based Materials Solutions Impregnated in Polymer Aerogels

**Figure 5:**
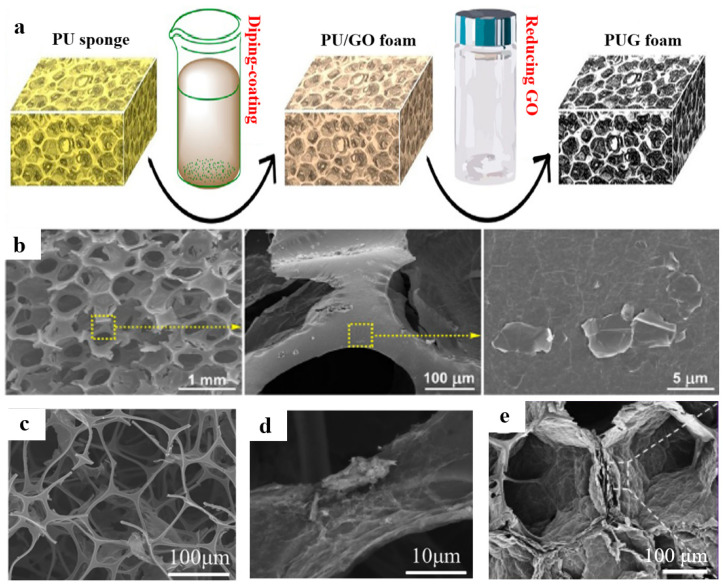
(**a**) Fabrication process of the PUG foam. (**b**) The SEM images of the PUG foam. Reproduced with permission from [64]. (**c**,**d**) The SEM images of ODA-RGO@MF foam. Reproduced with permission from [65]. (**e**) The microstructure of PFGA. Reproduced with permission from [66].

As shown in Figure 5a, Shen et al. [64] impregnated the commercial polyurethane (PU) sponge in the prepared GO suspension and dried it. During the drying process, the evaporation of water resulted in the self-assembly of GO sheets onto the surface of the PU skeleton. Finally, the ultra-light and compressible graphene/PU composite aerogel was obtained by reduction with hydrazine hydrate. As shown in Figure 5b, the PUG foam has a 3D porous structure, but the 3D skeleton is rough, which indicates that the RGO sheets had been successfully assembled around the PU skeleton. Chen et al. [65] prepared octadecylamine-grafted reduced GO@melamine foam (ODA-RGO@MF) by impregnating the MF in ODA-RGO. As shown in Figure 5c, the porous structure was well retained after coating with ODA-RGO, showing that ODA-RGO@MF perfectly inherited the 3D structure of MF, and no obvious damage occurred during the impregnating process. At the same time, the accumulation of irregular ODA-RGO on the MF skeleton can be observed in Figure 5d, and the surface roughness of the foam skeleton increases significantly. Pang et al. [66] prepared phenolic resin/graphene aerogel (PFGA) for sound-absorbing materials by impregnating phenolic foam in GO solution and water-plastic foaming. As shown in Figure 5e, RGO sheets with porous structure are anchored on a 3D phenolic resin foam network.

The impregnation method is simple, and the structures of the composite aerogels are related to the impregnation times and impregnation concentration.

### 2.2. Liquid Blending Methods

The liquid blending methods have various compositing methods and strong inclusiveness, which are the most studied composite methods in the field of graphene-based materials/polymer composite aerogels at present.

#### 2.2.1. Graphene-Based Materials/Polymer Directly Blending

**Figure 6:**
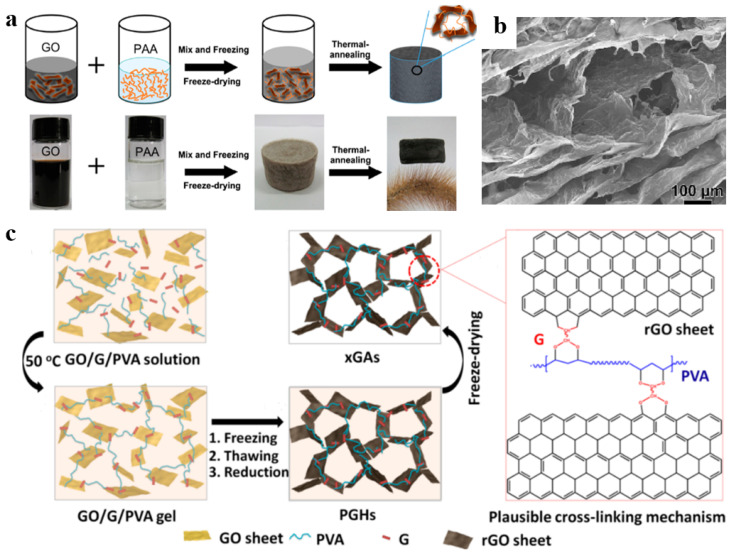
(**a**) Schematic illustration of the fabrication process of RGO/PI aerogel. (**b**) The SEM image of RGO/PI aerogel. Reproduced with permission from [71]. (**c**) Schematic showing the formation of graphene/PVA composite aerogels. Reproduced with permission from [74].

In this method, after mixing, reducing, and drying, the polymers and graphene-based materials solutions immediately form graphene-based materials/polymer composite aerogels [67,68,69,70]. As shown in Figure 6a, Qin et al. [71] prepared reduced GO/polyimide (RGO/PI) composite aerogels with ultra-high elasticity using a two-step method. Firstly, the polyimide precursor, polyamic acid (PAA), and GO dispersions were directly mixed. During the compositing process, PAA was anchored on the GO sheets using hydrogen bonding, which could limit the agglomeration effect of GO in the self-assembly process. After freeze-drying and heat treatment, the PAA was dehydrated to form polyimide, while GO was reduced and self-assembled into a highly porous structure, and then formed the RGO/PI composite aerogel. The remarkable layered skeleton of the RGO/PI composite aerogel helps to effectively transfer loads between RGO sheets and PI components under mechanical deformation, thereby improving mechanical properties (Figure 6b). Furthermore, polymers with active functional groups on the surface can also be crosslinked with GO by covalent bond or noncovalent bond, and then form composite aerogels [72,73]. Ye et al. [74] reported a series of lightweight and shape-recoverable graphene aerogels by cross-linking GO and polyvinyl alcohol (PVA) with an effective cross-linker glutaraldehyde (G) (Figure 6c). Aldehyde groups from G molecules reacted with hydroxyl groups of GO sheets and PVA chains by intermolecular acetalization. Therefore, PVA chains not only attached onto graphene surfaces through hydrogen bonds but also connected the neighboring graphene sheets by covalent cross-linking. Cross-linking interaction is beneficial to the structural stability of the composite aerogels.

#### 2.2.2. Graphene-Based Materials/Monomer Blending and Polymerization

**Figure 7:**
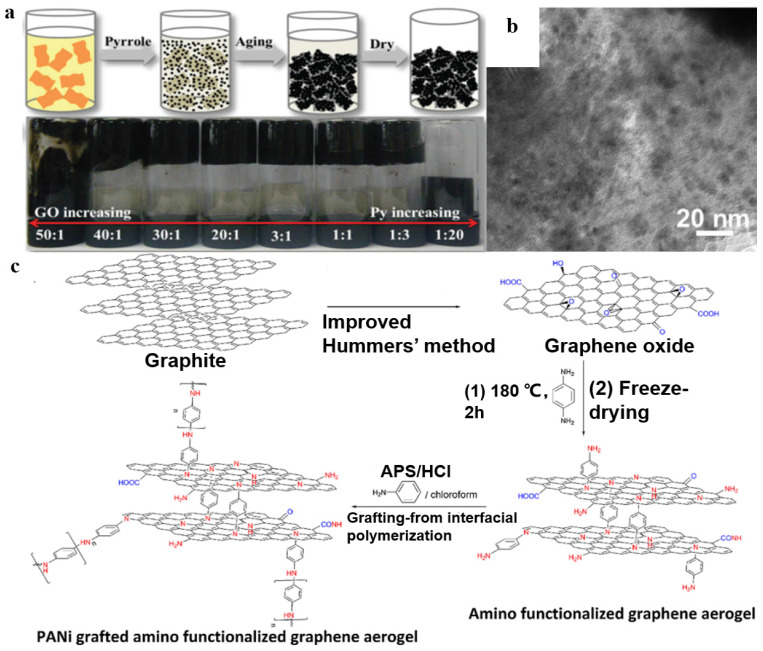
(**a**) Schematic illustration of the formation mechanism of the RGO/PPy aerogel. (**b**) The TEM image of RGO/PPy aerogel. Reproduced with permission from [78]. (**c**) Schematic procedure for synthesis of amino functionalized graphene aerogel grafted with polyaniline nanofibers. Reproduced with permission from [79].

In this method, polymer monomers and graphene or GO are polymerized to obtain graphene-based materials/polymer composite aerogels [75,76,77]. For example, Sun et al. [78] mixed GO dispersions and monomer pyrrole (Py) ultrasonically; the mixtures were aged at room temperature and formed hydrogels. During the aging process, pyrrole could reduce GO; it was oxidized and polymerized to form polypyrrole (PPy). Finally, graphene/PPy composite aerogel was obtained by freeze-drying (Figure 7a). As shown in Figure 7b, PPy in the form of plate-like nanoparticles with different sizes disorderedly anchors onto RGO nanosheets, which could be attributed to the polymerization of Py with different oxygen-containing groups that have different oxidizing abilities randomly attached on GO nanosheets. In addition, as shown in Figure 7c, Mahdavi et al. [79] prepared amino-functionalized graphene aerogel grafted with polyaniline nanofibers. Polyaniline nanofibers presented a small spherical structure on the surface of smooth aminated graphene aerogel. The formation of spheres may be due to both short PANI chains anchored on the amino groups that are on the surface of graphene aerogels and to heterogeneous nucleation.

#### 2.2.3. Graphene-Based Materials/Polymer Emulsion Blending

**Figure 8:**
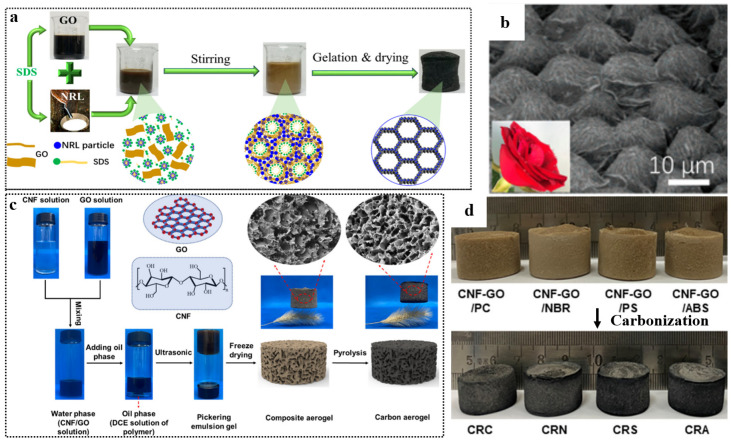
(**a**) Synthesis pathway of GA/NRL and the corresponding inner structure evolution. (**b**) Pictures of a rose petal and the surface morphology of GA/NRL. Reproduced with permission from [23]. (**c**) Schematic illustration of the preparation process of carbon aerogel. (**d**) Digital photos of the composite aerogel. Reproduced with permission from [80].

For traditional rubber latex, graphene-based materials/polymer composite aerogels can be obtained by direct emulsion blending with GNS or GO. For example, Zhang et al. [23] prepared a tough, ultralight (4.6 mg cm^−3^), highly compressible (>90%), and water-adhesive graphene/NRL hybrid aerogel (Figure 8a). As shown in Figure 8b, the NRL particles are wrapped by graphene layers to form sandwich-like cell walls with a rose-petal-like surface. In order to prepare graphene-based materials/polymer composite aerogels, some water-insoluble polymers can be dissolved in oily solvents and blended with graphene or GO in an emulsion. Liao et al. [80] mixed GO with cellulose nanofiber (CNF) to obtain a uniform aqueous phase dispersion, and then dissolved a series of polymers such as polycarbonate (PC), nitrile rubber (NBR), polystyrene (PS), and acrylonitrile butadiene styrene (ABS) into 1, 2 dichloroethane (DCE) to obtain an oil phase polymer DCE solution. The aqueous phase was then mixed with the oil phase to form an O/W Pickering emulsion gel. The gel was freeze-dried and carbonized, and graphene-based materials/polymer composite aerogel was formed (Figure 8c). The pictures of different polymer/graphene-based materials composite aerogels are shown in Figure 8d. However, the preparation of this composite aerogel is harmful to environment because of the use of oil solvents.

#### 2.2.4. Graphene-Based Materials/Polymeric Nanomaterials Blending

**Figure 9:**
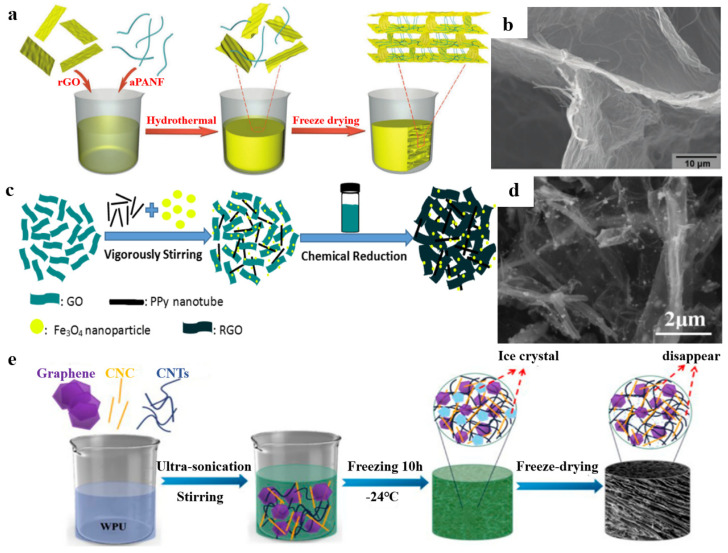
(**a**) The fabrication process of aPANF/GA. (**b**) The microstructure of aPANF/GA. Reproduced with permission from [83]. (**c**) Schematic fabrication process of GPFA. (**d**) The SEM images of GPFA. Reproduced with permission from [84]. (**e**) Schematic illustration of the fabrication of the CNTs/graphene/WPU/CNC composite aerogels. Reproduced with permission from [85].

Polymer nanomaterials are increasingly used as basic building blocks to prepare graphene-based materials/polymer composite aerogels due to the small size effect, surface effect and quantum effect of nanoparticles and the advantages of polymer materials [81,82]. For example, Cao et al. [83] reported nanofiber-reinforced graphene aerogels. There are three main steps (Figure 9a). First, the alkali-treated polyacrylonitrile nanofibers (aPAN) were dispersed uniformly in a GO aqueous solution by ultrasonic treatment. Then, hybrid hydrogel could be obtained by hydrothermal reaction. Finally, after dialysis treatment and freeze-drying, the nanofiber reinforced graphene aerogel could be obtained. As shown in Figure 9b, due to the surface treatment, the aPAN nanofibers have good compatibility with RGO sheets and are well embedded between the RGO sheets. Furthermore, Zhang et al. [84] prepared GO/PPy nanotubes/Fe_3_O_4_ aerogel through a simple one-step self-assembly process that used hydrothermal reduction (Figure 9c). First, a certain amount of PPy nanotubes and Fe_3_O_4_ nanoparticles were added into the GO solution, followed by vigorous stirring. Next, hydrazine hydrate was added into the mixed solution, and it was placed in the oven. Finally, the RGO/PPy Nanotube/Fe_3_O_4_ Aerogels could be obtained after freeze-drying. Figure 9d shows that the PPy nanotubes are uniformly attached on the surface of RGO sheets, but some are dispersed between RGO sheets; this is beneficial as it avoids the aggregation of RGO sheets during the hydrothermal reduction process. A combination of GO and nanocrystals also creates a good composite unit for aerogel preparation. For example, Zhai et al. [85] prepared the (CNTs)/graphene/waterborne polyurethane (WPU) composite aerogel (its fabrication process is illustrated in Figure 9e). The amphiphilic cellulose nanocrystals (CNC) are helpful in stabilizing the carbon nanofillers and forming a stable CNTs/graphene dispersion. More importantly, CNC is beneficial for establishing a fragile conductive network. The structural scale of polymer nanomaterials is matched with graphene-based materials, which are easier to composite.

#### 2.2.5. Liquid Blending and 3D Printing

**Figure 10:**
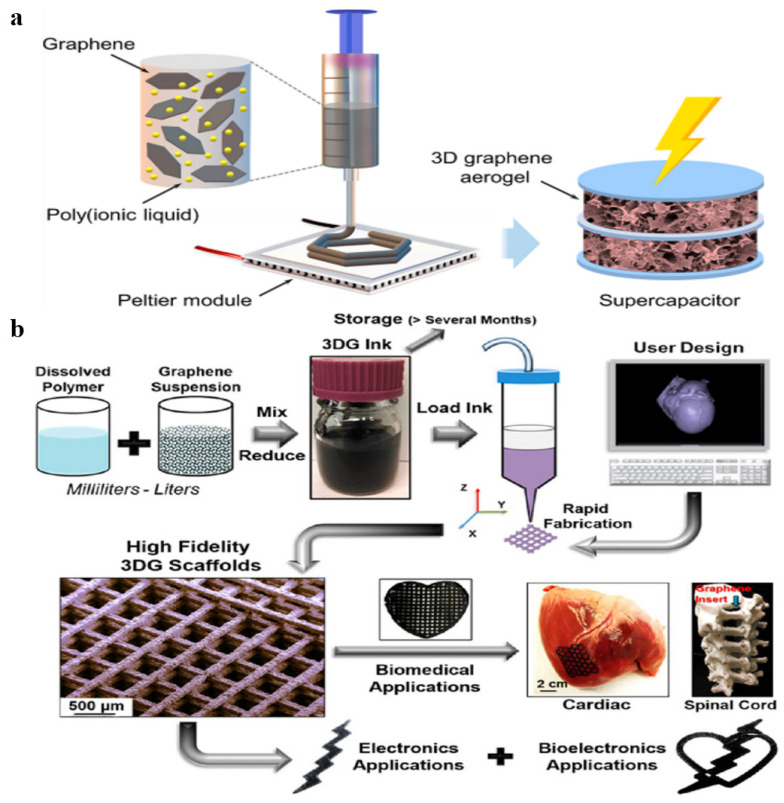
(**a**) The fabrication process of graphene/imidazolium-based poly(ionic liquid)s composite aerogel. Reproduced with permission from [86]. (**b**) Schematic illustration of the preparation process of graphene/PLG composite aerogel. Reproduced with permission from [88].

In addition to the traditional liquid phase blending methods, 3D printing technology as a preparation method for graphene-based materials/polymer composite aerogels has seen increasing interest in recent years. As shown in Figure 10a, Choudhury et al. [86] prepared graphene/imidazolium-based poly(ionic liquid)s composite aerogel that uses 3D freeze printing. A range of polymers have been used to prepare stable graphene dispersions in many different solvents, and even water [87]. In this work, the presence of poly(ionic liquid)s not only stabilized and enhanced the characteristics of ejectable graphene nanoinks; it also acted as a binder for the construction graphene nanosheets into a 3D graphene/polymer composite aerogel. Jakus et al. [88] reported a 3D printable graphene/polylactide-co-glycolide (PLG) composite aerogel (Figure 10b). On the one hand, PLG acted as binder in solvent-based 3D graphene ink. On the other hand, PLG has the advantage of biocompatibility and biodegradability. Therefore, the final composite aerogel played an important role in medicine, bioelectronics, and so on. The 3D printing method is suitable for preparing high-precision graphene-based materials.

### 2.3. Melt Blending Methods

At present, there are few studies on the preparation of graphene-based materials/polymer composite aerogels by melt blending. This method first melts the polymer and graphene to obtain a liquated composite, and then uses injection foaming technology or supercritical carbon dioxide (scCO_2_) foaming technology to obtain a graphene/polymer composite aerogel [89,90,91,92,93]. For example, Xiao et al. [94] fabricated composites that combined polystyrene (PS) and thermally reduced graphene using melting blending. Then, PS/graphene composite foams were prepared using the scCO_2_ foaming process. The melt-blending method has strict requirements for the processing temperature and is not easy to scale up.

## 3. Properties and Applications of Graphene-Based Materials/Polymer Composite Aerogels

When combining the excellent properties of graphene with different polymers, graphene-based materials/polymer composite aerogels with “1 + 1 > 2” effects in mechanical, electrical, electrochemical, thermal, and adsorption properties and other properties will be obtained. These composite aerogels have shown great potentials in many fields, such as wearable sensors, electrode materials, thermal management, adsorbing materials, etc.

### 3.1. Mechanical Properties and Applications

**Figure 11:**
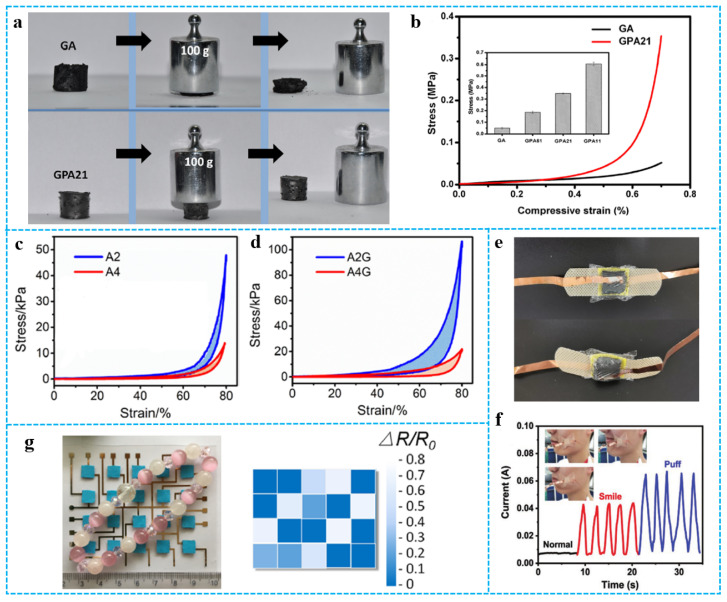
(**a**) Digital images showing compressibility of pure GA and GPA21. (**b**) The compressive stress-strain curves of GA and GPA21 at the maximum strain of 70%, inset is the average compressive strength of GA and GPAs. Reproduced with permission from [24]. (**c**,**d**) Stress-strain curves of compression-decompression tests on A2/A4 and A2G/A4G. Reproduced with permission from [99]. (**e**) Digital photo of the assembled CNF/CNT/RGO carbon aerogels sensor. (**f**) Current signals from facial expressions (normal, smile, and puff). Reproduced with permission from [106]. (**g**) Bracelets on flat PDMS/graphene aerogel sensor arrays and the signal output. Reproduced with permission from [99].

The introduction of polymers will improve the shortcomings of poor strength and toughness of graphene aerogels to a certain extent [95,96,97]. Ye et al. [24] prepared 3D hierarchical graphene/PPy aerogel (GPA). As shown in Figure 11a, pure graphene aerogel (GA) could be easily squeezed into a pellet under pressure, although it recovered from strain by almost as much as 50%. Conversely, the GPAs showed better mechanical strength and press resistance. For instance, GPA21 monolith (~20 mg) could support over 5000 times of its own weight without obvious destruction. The mechanical property of the aerogels was presented through the compressive stress-strain curves (Figure 11b). GPA21 had a compressive strength of ~0.35 MPa at 70% compression strain, which is 7-fold higher than that of pure GA (0.05 MPa). The average compressive strengths for each sample are shown in the inset of Figure 10b, with the increase of the mass ratio of the PPy nanotube (PNT) to GO from 1:5 to 1:2 and 1:1, and the obtained corresponding aerogels, GPA51, GPA21, and GPA11, demonstrated improved compressive strengths of 0.19, 0.35, and 0.61 MPa, respectively. The enhancement effect of PNTs and the strong interfacial interactions between graphene sheets and PNTs are helpful to improve GPA. Similarly, graphene also improved the strength of pure polymer aerogel [98]. In Figure 11c,d, the compressive strength of RGO/polyorganosiloxane (PDMS) aerogel (A2G) is much higher than that of the initial PDMS aerogel (A2), which may be due to the cross-links of RGO nanosheets with polyvinylmethyldimethoxysilane (PVMDMS) polymers [99]. Graphene/polymer composite aerogels have great potential as pressure and strain sensors and play an important role in wearable and integrated sensors [100,101,102,103,104,105]. The sensing performance of the sensor is closely related to the elasticity and mechanical strength of graphene-based materials/polymer composite aerogels. For example, the elasticity of composite aerogels is important to the sensitivity of the sensor. Composite aerogels with high mechanical strength are suitable for large strain sensing. Liu et al. [106] assembled CNF/CNT/RGO-3 carbon aerogels as devices that could assess the sensing performance for wearable sensors (Figure 11e). Such sensors could capture different current signals from facial expressions, such as smiles and puffs (Figure 11f). In addition, as shown in Figure 11g, Zu et al. [99] prepared a flexible 4 × 5 RGO/PDMS aerogel-based sensing array. A bracelet was placed on the flat sensing array to determine the strain (or pressure) distribution generated by the loading.

### 3.2. Electrical Properties and Applications

**Figure 12:**
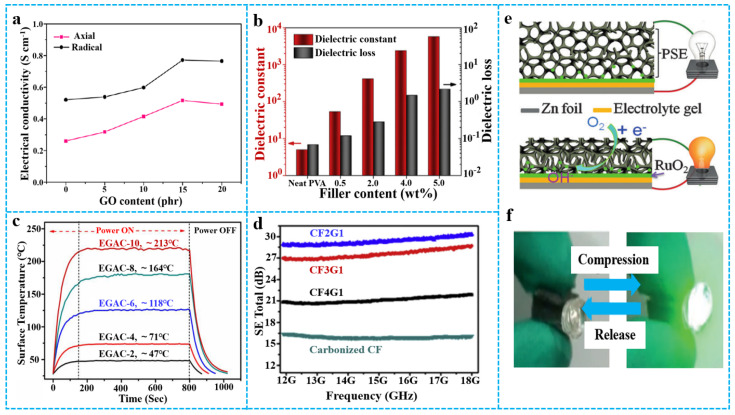
(**a**) Electrical conductivity of PI/graphene aerogels in two directions. Reproduced with permission from [109]. (**b**) Summary of dielectric properties of PVA/graphene aerogel at 1 kHz. Reproduced with permission from [110]. (**c**) Temperature-time response curves of epoxy/graphene-based aerogel composites with different graphene contents. Reproduced with permission from [112]. (**d**) EMI shielding performance of CF/RGO aerogel (5 mm) with different mass ratios in the frequency range from 12 to 18 GHz. Reproduced with permission from [25]. (**e**) Schematic structure and working principle of tactile sensing battery under compression. (**f**) Loading of a compressive force onto a tactile sensing battery increases its output power, illuminating the green LED. Reproduced with permission from [26].

Due to the addition of graphene-based materials, the electrical properties of polymer-based aerogels have been greatly improved [107,108]. The conductive path of structure and composite uniformity of graphene-based materials/polymer composite aerogels determine the conductivity of the material. For example, Jiang et al. [109] reported graphene/PI composite aerogels containing different contents of GO. As shown in Figure 12a, the electrical conductivity of graphene/PI composite aerogels was gradually enhanced while the original GO content increased. This occurred because RGO had good conductivity, forming effective conductive paths in the material. Wang et al. [110] prepared graphene/PVA composite aerogels with excellent dielectric properties by a two-step method. Figure 12b shows the dielectric constant and dielectric loss of graphene/PVA composite aerogels with different graphene contents. The highest dielectric constant achieved was 5720 at a graphene content of 5 wt%, equivalent to almost 1150 times that of the neat PVA. The high dielectric constants may be due to the full exfoliation and dispersion of RGO sheets in the polymer matrix. However, the enhanced dielectric constants were always at the expense of similar increases in loss, which was attributed to the motion of free charge carriers resulting from the formation of continuous conductive networks. Due to excellent electrical conductivity and thermal conductivity, graphene aerogel can be composited into the polymer matrix as a functional filler, thereby achieving application value in electrothermal field [111]. Yang et al. [112] reported graphene aerogel/epoxy resin nanocomposites (EGAC) with excellent electrothermal properties. Figure 12c shows that the steady-state surface temperature of EGAC increased as the density of the graphene aerogel increased. The improved Joule heating performances of the aerogel composites are attributed to the 3D morphology and original structural properties of graphene scaffolds. The electromagnetic shielding performance of the composite material depends on electrical performance and magnetic performance. However, the spatial distribution of graphene aerogels plays an important role in the mechanical and electromagnetic shielding properties of polymer composites. As seen from Figure 12d, cellulose fiber/RGO (CF/RGO) aerogels are closely related to the loading of RGO at a fixed frequency. For example, at 18 GHz, the shielding effectiveness (SE) total of the composite aerogel of that weight ratio of CF and GO was 2:1, and when treated at 600 °C under argon (CF2G1-600A), is almost twice as much as the pure cellulose fiber aerogel [25]. The improved electromagnetic interference (EMI) SE was mainly ascribed to increased RGO loading and enhanced electrical conductivity, which had a substantial connection with EMI shielding performance [113]. A higher RGO loading leads to stronger interaction with the incident electromagnetic waves, achieving improved SE [114]. In addition, Wang et al. [26] reported graphene/polyurethane composite aerogels for applications in new energy management devices, such as tactile sensing batteries, that can perform environmental pressure sensing (Figure 12e,f).

### 3.3. Electrochemical Properties and Applications

**Figure 13:**
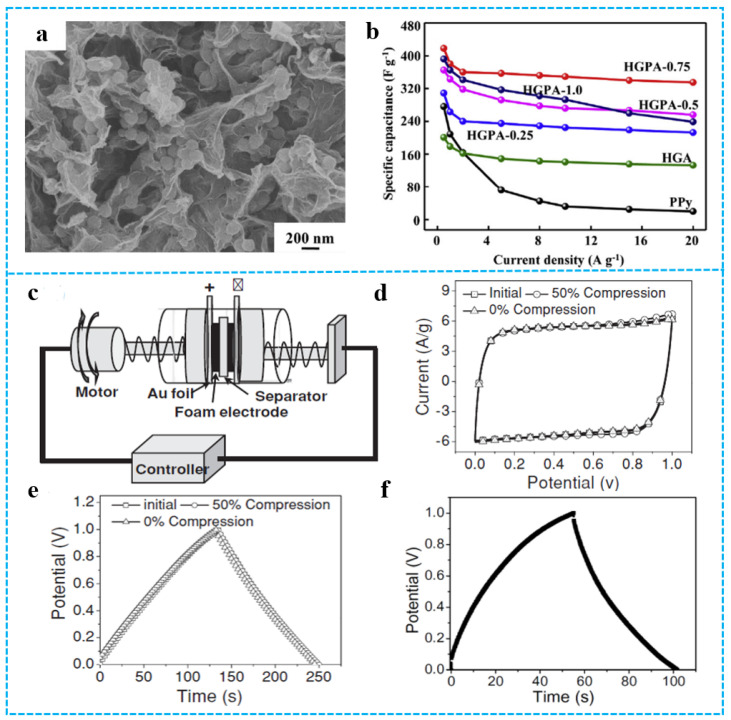
(**a**) The SEM image of HGPA-0.75. (**b**) Capacitive performances of various electrodes in 1.0 M KOH electrolyte: capacitance retention at different current densities. Reproduced with permission from [121]. (**c**) Schematic illustration of PPy-G-based supercapacitor devices. (**d**) CVs of the compressible supercapacitor cells based on PPy-G foam electrodes under 0% and 50% compression for one cycle. The scan rate is 30 mV/s. (**e**) The corresponding galvanostatic charge-discharge curves at a current of 1.5 A g^−1^ under 0% and 50% compression. (**f**) The galvanostatic charge-discharge curves of two-electrode 3D graphene supercapacitor at a current of 1.5 A/g. Reproduced with permission from [41].

The conductivity of graphene can reach (6000 S/cm), which makes it possible to prepare composite materials with excellent electrochemical properties. However, the serious restacking of the 2D graphene layer during the manufacturing process leads to lower conductivity. The 3D graphene-based materials aerogel with interconnected network and abundant porous structure can effectively prevent the accumulation of 2D graphene layers, showing good and stable electrochemical performance. For conductive polymers such as polyaniline [115,116], PPy [117,118,119], and polythiophene [120], they can play a synergistic role with graphene aerogels and greatly improve the electrochemical properties of composite aerogels due to their good electrical conductivity, easy synthesis, high redox pseudocapacitive charge storage, etc. He et al. [121] fabricated holey graphene/PPy composite aerogels (HGPAs) with 3D hierarchical structure by using freeze-drying technology. Figure 13a shows that PPy nanoparticles embedded between the holey graphene (HG) nanosheets further limit the restacking of HG nanosheets. The pore structure of composite aerogels was important to shorten the ion pathway and enhance ion kinetics. HGPA-0.75 composite aerogel electrode showed high specific capacitance (418 F g^−1^) at a current density of 0.5 A g^−1^ (Figure 13b). The size and composite ratio of conductive polymer influences electrochemical performance. For example, the composite aerogel electrode with the larger PPy nanoparticles have more excellent capacitance performance [121]. Graphene-based materials/polymer composite aerogels with excellent electrochemical properties can be used as electrode materials for supercapacitors [122]. As an example, Zhao et al. [41] used PPy as a mediator and developed a unique strategy for in situ formation of PPy-graphene (PPy-G) foam. Figure 13c demonstrated a highly compression-tolerant graphene-based supercapacitor. As shown in Figure 13d, owing to the conductivity and excellent mechanical strength of PPy-G foam electrodes, the cyclic voltammetry (CV) curves of the assembled capacitor retained a rectangular shape with ideal capacitive behavior. No obvious change was observed in the CVs of the compressed supercapacitors with a 50% applied strain. The constant current charge-discharge curves of PPy-G foam electrode and graphene aerogel electrode supercapacitors at 1.5 A/g current are shown in Figure 13e,f. The specific capacitance calculated from the discharge slopes was ca. 350 F/g, which was much higher than that of both compact PPy film (ca. 50 F/g) and pure 3D graphene (ca.151 F/g). This enhanced the capacitance of PPy-G because of the synergetic function of PPy and graphene aerogel.

### 3.4. Thermal Properties and Applications

**Figure 14:**
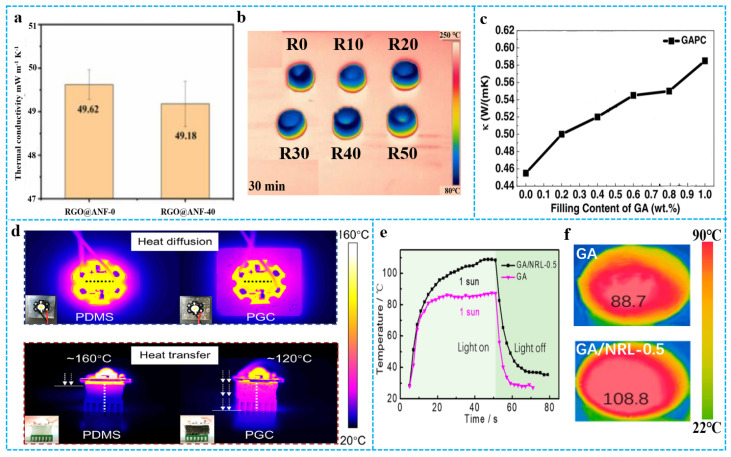
(**a**) Thermal conductivity of RGO@ANF-0 and RGO@ANF-40. (**b**) Thermal infrared images of all aerogels with the temperature of the warm table at 250 °C, R0 to R50 represent RGO@ANF-0 to RGO@ANF-50 respectively. Reproduced with permission from [123]. (**c**) The thermal conductivities of GAPC with different GA contents. Reproduced with permission from [124]. (**d**) Infrared images of an LED lamp with PDMS and a PGC for heat dissipation and infrared images of a LED lamp with PDMS and a PGC for heat transfer. Reproduced with permission from [126]. (**e**) Temperature changing curves of “light on” and “light off” under 1 sun. (**f**) Solar-thermal behavior of GA/ NRL-0.5 and GA under 1 sun. Reproduced with permission from [23].

Graphene aerogels can be used in thermal insulation due to their low density and high porosity. The components and pore structures of graphene-based materials composite aerogels are important to thermal insulation. Figure 14a shows that in the thermal conductivity of RGO@aramid nanofiber−-0 (RGO@ANF−0) and RGO@ANF−40, the 0 and 40 refer to the ANF ratio. It can be found that the addition of ANF weakens the heat transfer ability between graphene, and the aerogel structure increased the heat transfer path, so the thermal conductivity of RGO@ANF-40 is lower. As shown in Figure 14b, RGO@ANF-0 to RGO@ANF-40 aerogels were placed on the warm table at 250 °C for 30 min, the surface of RGO@ANF-0 had the highest temperature [123]. Graphene has excellent thermal conductivity and is an ideal filler for preparing composite materials with great thermal properties. Zhang et al. [124] prepared graphene/PDMS composite aerogels with excellent thermal conductivity using the impregnation method. As shown in Figure 14c, the thermal conductivity of GAPC rose with increased GA loading from 0.2 wt% to 1 wt% and reached 0.58 W/(mK) at 1 wt%. Benefitting from the high thermal conductivity of aerogel, graphene-based materials composite aerogel could relieve the urgent thermal management demands in electronic devices [125]. For example, the graphene/PDMS aerogel and pure PDMS were used as heat diffusion and heat transfer material for a high-power LED lamp [126]. Figure 14d shows that the introduction of graphene aerogel improves the thermal management ability of pure PDMS. In addition, graphene/polymer composite aerogels exhibit broad optical absorption over the visible and near-infrared (NIR) regions and have been employed as efficient photothermal agents for photothermal therapy and solar desalination. As an example, Zhang et al. [23] reported graphene/ NRL aerogel with good solar–thermal conversion efficiency. As shown in Figure 14e,f, IR imaging was employed to monitor the temperature of GA/NRLs upon irradiation with a simulated solar beam (1 sun = 1000 W m^−2^). Upon irradiation (1 sun), the surface temperature of GA/NRL−0.5 rapidly increased to 108.8 °C within 50 s, which is much higher than that of GA (88.5 °C). The enhanced solar–thermal conversion property of GA/NRL-0.5 resulted from the broad optical absorption of the RGO skeleton and the light scattering from the numerous papillae. Furthermore, nanowrinkles extended the optical path within the aerogel and captured a significant amount of light. Natural rubber particles among the RGO sheets could effectively reduce the heat dissipation between graphene networks.

### 3.5. Adsorption Properties and Applications

**Figure 15:**
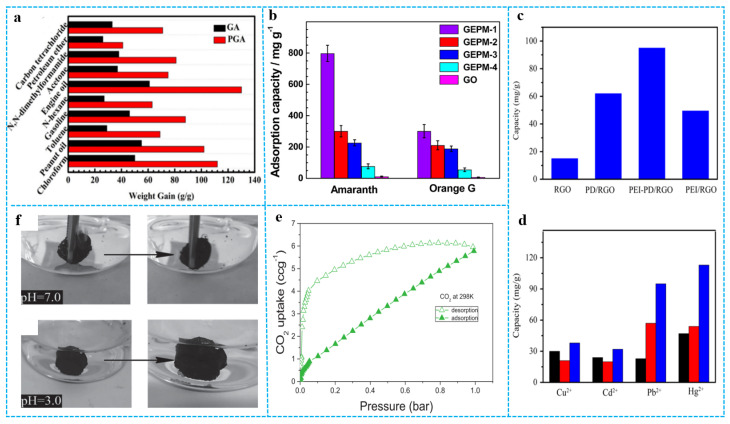
(**a**) Absorption capacities of GA and PGA for various oil and organic solvents. Reproduced with permission from [133]. (**b**) Adsorption capacities of GEPMs for amaranth and orange G by GO-PEI porous material (GEPM) and GO. Reproduced with permission from [134]. (**c**) Adsorption capacity of Pb^2+^ on RGO, PD/RGO, PEI-PD/RGO, and PEI/RGO aerogels. (**d**) Comparison of adsorption capacity of Cu^2+^, Cd^2+^, Pb^2+^, and Hg^2+^ on active carbon (black), PEI/RGO (red), PE1-PD/RGO (blue) aerogels. Reproduced with permission from [135]. (**e**) CO_2_ adsorption and desorption of chitosan-grafted GO aerogel at 298 K. Reproduced with permission from [136]. (**f**) The adsorption of chloroform by the *ss*-GF in water of pH 7.0 and the desorption of chloroform by the *ss*-GF in water of pH 3.0. Reproduced with permission from [137].

Graphene aerogels can be used as adsorption materials because of their high specific surface areas, high porosities, and hydrophobicity. Combining them with functional polymers can further improve the adsorption capacity and efficiency [127,128,129,130,131,132]. For example, Zhang et al. [133] reported a dimethyldiallylammonium chloride acrylamide polymer (P(AM-DMDAAC))/graphene aerogel (PGA) with high adsorption capacity and high hydrophobicity by one-step hydrothermal treatment of doping P(AM-DMDAAC) into graphene aerogel. P(AM-DMDAAC) has been widely used in oil extraction, papermaking, and flocculation because of its high charge density, alkali resistance, and temperature resistance. As shown in Figure 15a, PGA had higher adsorption capacity for various oils and organic solvents as compared with pure graphene aerogel (GA). The adsorption capacities of PGA ranged from 40 to 130 times of its initial weight. Graphene/polymer composite aerogels can rapidly diffuse dye molecules to adsorption sites due to their high specific surface areas and cross-linked network structures, which greatly improves the adsorption capacity and adsorption efficiency of dyes and provides a new idea for the treatment of dye-containing wastewater. For instance, Sui et al. [134] prepared the 3D graphene/polyethylenimine (PEI) composite aerogel, which shows an ultra-high adsorption capacity (800 mg/g) for the acid dye amaranth; this is much higher than other carbon materials. For comparison, the adsorption capacities of pure GO powder for amaranth and orange G were calculated to be 11 and 7 mg/g, respectively (Figure 15b). Dong et al. [135] prepared polyethylenimine-polydopamine/RGO (PEI-PD/RGO) aerogel through a hydrothermal method, which achieved a high surface area of up to 373 m^2^/g. In this work, polydopamine (PD) was utilized as an interlayer to bind GO and (PEI) brushes. The uniform and well-controlled coating of an ultrathin PD layer on GO not only enhanced the hydrophilicity of GO sheets, but also provided abundant active sites for further grafting of PEI. The adsorption capacity of the RGO, PD/RGO, PEI/RGO, and PEI-PD/RGO aerogels to Pb^2+^ were first researched as shown in Figure 15c. Among them, PEI–PD/RGO aerogel exhibited the highest capacity (95 mg/g) to Pb^2+^. This indicated that the synergistic effect between PEI and RGO greatly improved the adsorption performance. Figure 15d presents the adsorption capacity of Cu^2+^, Cd^2+^, Pb^2+^, and Hg^2+^ on PEI/RGO aerogel and PEI–PD/RGO aerogel and commercialized active carbon. The capacities of PEI-PD/RGO aerogel for Cu^2+^, Cd^2+^, Pb^2+^, and Hg^2+^ are 38, 32, 95, and 113 mg/g, respectively. Graphene/polymer composite aerogels also show the gas-absorption ability. Hsan et al. [136] demonstrated a superficial, environmentally friendly, and sustainable development of chitosan (CS)-grafted GO aerogels for adsorption of CO_2_ gas. As shown in Figure 15e, the maximum CO_2_ adsorption that was recorded on the surface of the aerogels was 11.35 mg g^−1^ (0.257 mmol g^−1^) as the pressure was increased to 1 bar. Generally, organic solvents or dyes adsorbed in graphene composite aerogels require external forces to achieve desorption, such as extrusion or heating evaporation. In recent years, researchers have invented new methods to achieve the adsorption and desorption of adsorbates under external stimuli (pH, temperature, and electricity). For example, Zhu et al. [137] prepared smart graphene/polymer composite aerogels (ss-GF) by grafting amphiphilic block copolymer (P2VP-b-PHA) on the surface of GA. Figure 15f shows that the ss-GF absorbed chloroform quickly due to hydrophobicity and oleophilicity of the ss-GF in the neutral aqueous medium. Because the high repulsive force between the polar and nonpolar materials and the polar and the protonated P2VP chains were gradually wetted by the polar acidic water, nonpolar chloroform was expelled from the ss-GF drop by drop. This data suggests that the ss-GF were able to adsorb and desorb the underwater organic solvents by varying the medium pH.

### 3.6. Other Properties and Applications

**Figure 16:**
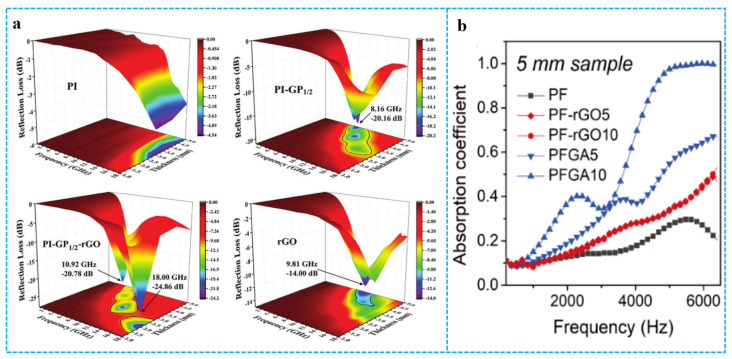
(**a**) 3D reflection loss maps of PI, PI-GP_1/2_, PI-GP_1/2_-rGO and RGO composite foams. Reproduced with permission from [138]. (**b**) The sound-absorption coefficients of PF, PF-RGO and PFGA at 5 mm thickness. Reproduced with permission from [57].

Graphene-based materials/polymer composite aerogels rely on multiple reflections between pore structures to achieve attenuation of incident electromagnetic waves or by controlling the impedance gradient of the material skeleton, which can make a big difference in absorbing materials. As an example, Pu et al. [138] exactly designed a polyimide/graphene composite aerogel with a multi-stage impedance gradient structure. As shown in Figure 16a, in the entire frequency range, the microwave absorption (MA) performance of pure PI is unsatisfactory. The pure RGO aerogel possessed minimum reflection loss (RL_min_) value of −14 dB when the absorber thickness and frequency are 5 mm and 9.81 GHz, respectively. Its effective bandwidth (RL < −10 dB) was 3.73 GHz (8.16–11.89 GHz). After being combined with RGO and PI_1/2_, the RL_min_ value of the PI-GP_1/2_ foam could decrease to −20.15 dB at 8.16 GHz when the thickness is 5 mm. The RL_min_ value at 18.0 GHz of PI-GP_1/2_-RGO with a thickness of 2 mm was −24.86 dB, and the effective bandwidth was improved to 3.97 GHz (14.03–18 GHz). Comparison with PI-GP_1/2_ and pure RGO foams, the obvious enhancement in absorption capacity and bandwidth can be attributed to the successful construction of the three-level impedance gradient in the foam. In addition, graphene/polymer composite aerogels can be applied in sound absorption [139]. Pang et al. [57] reported a cellular acoustic absorber with ultrahigh efficiency in acoustic absorption. The graphene acoustic absorber was integrated by self-standing ultrathin graphene drums and PF frame by the hydroplastic foaming method. Figure 16b shows that the absorption coefficient of PFGA was much higher than that of the PF, in both the high and low frequency range. The sound-absorption ability of PFGA continuously increased with GO content. Benefitting from the synergistic dissipation of ultrathin graphene and porous cavity, the acoustic absorber exhibits remarkable enhancement by ~320% of average absorption from 200 to 6000 Hz. The multi-layer structure of the composite aerogels is crucial to the wave absorption/sound absorption performance. In addition, fiber is a good composite filler for composite aerogels applied in the wave absorption/sound absorption field. The fiber itself has wave- and sound-absorption properties, which is conducive to the multiple reflection and scattering of sound waves and electromagnetic waves in the material.

## 4. Summary and Outlook

Graphene-based materials/polymer composite aerogels have attracted increasing amounts of attention in recent years. In this paper, the research progress of graphene-based materials/polymer composite aerogels is systematically reviewed, including the preparation methods, structures, interactions, properties, and applications.

From the perspective of preparation methods, the impregnation methods are simple and easy to scale up. These methods may be the most likely way to achieve industrial mass production. However, these methods have a limited range of structural variation and material properties. At present, the liquid phase blending methods are commonly used because these methods can easily extend to many types of polymers and the prepared composite aerogels show several different 3D networks and properties. Therefore, more and more advanced composite aerogels with unique properties could be prepared using these methods. In terms of structures and interaction, the structures of graphene-based materials/polymer composite aerogels prepared using the impregnation method are often a multi-layer structure with a layer of graphene or polymer coating over a 3D network skeleton. In the liquid phase blending methods, the graphene-based materials solutions are more uniformly compounded with the polymers than in the other methods. In the structure of composite aerogels, polymers often act as a bridge to reduce the stacking of graphene sheets. Different kinds of composite structures and interactions can be achieved using the liquid phase blending methods.

First, in terms of properties and applications, the introduction of polymers more effectively improves the mechanical properties of materials than pure graphene aerogels do, and they show superior results in sensor applications. Second, in terms of electrical properties and applications, most polymers are nonconductive. The addition of graphene aerogels can endow them with excellent electrical properties. At the same time, graphene aerogels can play a synergistic role with conductive polymers and show significant application effects in supercapacitors, electromagnetic shielding, and compressible batteries. Third, in terms of thermal properties and applications, graphene aerogel itself has thermal insulation and photothermal properties. The addition of some polymers can improve these properties. Thus, graphene-based materials/polymer composite aerogels can be applied in demanding fields. In addition, since graphene has excellent thermal conductivity, it can be used as a filler to prepare graphene-based materials/polymer composite aerogels with high thermal conductivity, and the composite aerogels show application prospects in thermal management. Fourth, in terms of adsorption performance and application, graphene aerogels can be compounded with functional polymers, and have shown more excellent results in oil absorption, dye and gas adsorption, heavy metal ion adsorption and intelligent adsorption and desorption. Finally, graphene-based materials/polymer composite aerogels also show the performance and great application prospects in advanced functions, such as microwave absorption and sound absorption. Ultimately, for many mechanical and functional properties, graphene-based materials/polymers composites can offer better performance than a single aerogel or foam due to their synergistic effects.

In summary, the future research direction of graphene-based materials/polymer composite aerogels will be directed toward the precise design and large-scale preparation of composite materials with multidimensional properties and better performance.

## Data Availability

The data presented in this study are available upon request from the corresponding author, based on reasonable requirements.
